# Penta­aqua­(4,6-dihy­droxy­benzene-1,3-disulfonato-κ*O* ^1^)zinc penta­hydrate

**DOI:** 10.1107/S1600536812012317

**Published:** 2012-03-28

**Authors:** Zhi-Biao Zhu, Shan Gao, Seik Weng Ng, Edward R. T. Tiekink

**Affiliations:** a Key Laboratory of Functional Inorganic Material Chemistry, Ministry of Education, Heilongjiang University, Harbin 150080, People’s Republic of China; b Department of Chemistry, University of Malaya, 50603 Kuala Lumpur, Malaysia; c Chemistry Department, Faculty of Science, King Abdulaziz University, PO Box 80203 Jeddah, Saudi Arabia

## Abstract

The Zn^II^ atom in the title complex, [Zn(C_6_H_4_O_8_S_2_)(H_2_O)_5_]·5H_2_O, is coordinated by five water mol­ecules and an O atom of a 4,6-dihy­droxy­benzene-1,3-disulfonate dianion. The coord­ination geometry is distorted octa­hedral, with the Zn—O_sulfonate_ bond relatively long compared to the Zn—O_water_ bonds. The coordinated and lattice water mol­ecules inter­act with each other and with the hy­droxy groups and sulfonate ligand through O—H⋯O hydrogen bonds, generating a tightly held three-dimensional network.

## Related literature

For related structures, see: Xie *et al.* (2010 ▶); Bakirci *et al.* (2006 ▶).
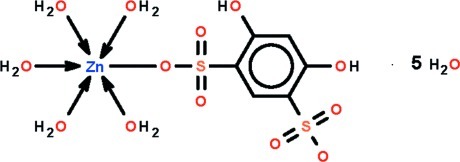


## Experimental

### 

#### Crystal data

[Zn(C_6_H_4_O_8_S_2_)(H_2_O)_5_]·5H_2_O
*M* *_r_* = 513.74
Triclinic, 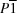

*a* = 7.1479 (3) Å
*b* = 11.8929 (5) Å
*c* = 12.2044 (6) Å
α = 109.368 (1)°
β = 104.690 (1)°
γ = 92.953 (1)°
*V* = 936.27 (7) Å^3^
*Z* = 2
Mo *K*α radiation
μ = 1.62 mm^−1^
*T* = 293 K
0.21 × 0.17 × 0.14 mm

#### Data collection

Rigaku R-AXIS RAPID IP diffractometer
Absorption correction: multi-scan (*ABSCOR*; Higashi, 1995 ▶) *T*_min_ = 0.747, *T*_max_ = 0.820
9270 measured reflections
4257 independent reflections
3883 reflections with *I* > 2σ(*I*)
*R*_int_ = 0.019

#### Refinement

*R*[*F* ^2^ > 2σ(*F* ^2^)] = 0.032
*wR*(*F* ^2^) = 0.091
*S* = 1.06
4257 reflections
310 parameters
32 restraints
H atoms treated by a mixture of independent and constrained refinement
Δρ_max_ = 0.67 e Å^−3^
Δρ_min_ = −0.35 e Å^−3^

### 

Data collection: *RAPID-AUTO* (Rigaku, 1998 ▶); cell refinement: *RAPID-AUTO*; data reduction: *CrystalClear* (Rigaku/MSC and Rigaku, 2002 ▶); program(s) used to solve structure: *SHELXS97* (Sheldrick, 2008 ▶); program(s) used to refine structure: *SHELXL97* (Sheldrick, 2008 ▶); molecular graphics: *X-SEED* (Barbour, 2001 ▶) and *DIAMOND* (Brandenburg, 2006 ▶); software used to prepare material for publication: *publCIF* (Westrip, 2010 ▶).

## Supplementary Material

Crystal structure: contains datablock(s) global, I. DOI: 10.1107/S1600536812012317/xu5482sup1.cif

Structure factors: contains datablock(s) I. DOI: 10.1107/S1600536812012317/xu5482Isup2.hkl

Additional supplementary materials:  crystallographic information; 3D view; checkCIF report

## Figures and Tables

**Table 1:** Selected bond lengths (Å)

Zn—O1	2.5415 (15)
Zn—O1*w*	1.9950 (15)
Zn—O2*w*	1.9460 (18)
Zn—O3*w*	1.9671 (17)
Zn—O4*w*	1.9588 (17)
Zn—O5*w*	2.2355 (17)

**Table 2:** Hydrogen-bond geometry (Å, °)

*D*—H⋯*A*	*D*—H	H⋯*A*	*D*⋯*A*	*D*—H⋯*A*
O7—H7⋯O9*w*^i^	0.83 (1)	1.79 (1)	2.612 (2)	170 (3)
O8—H8⋯O8*w*^ii^	0.83 (1)	1.84 (1)	2.664 (2)	172 (3)
O1*w*—H11⋯O2	0.84 (1)	2.09 (2)	2.852 (2)	151 (3)
O1*w*—H12⋯O7^iii^	0.83 (1)	2.09 (1)	2.923 (2)	175 (3)
O2*w*—H21⋯O6^iv^	0.83 (1)	2.22 (1)	3.021 (3)	162 (3)
O2*w*—H22⋯O7*w*^iii^	0.83 (1)	1.88 (1)	2.710 (3)	171 (4)
O3*w*—H31⋯O6*w*^iv^	0.84 (1)	2.01 (2)	2.765 (2)	149 (3)
O3*w*—H32⋯O10*w*^v^	0.83 (1)	1.79 (1)	2.618 (3)	178 (5)
O4*w*—H41⋯O6*w*^vi^	0.83 (1)	1.98 (1)	2.774 (2)	160 (3)
O4*w*—H42⋯O7*w*^vii^	0.84 (1)	1.99 (1)	2.802 (3)	164 (3)
O5*w*—H51⋯O4^v^	0.83 (1)	2.00 (1)	2.833 (3)	176 (4)
O5*w*—H52⋯O6^viii^	0.84 (1)	1.97 (1)	2.802 (3)	170 (3)
O6*w*—H62⋯O1	0.84 (1)	2.08 (1)	2.903 (2)	168 (3)
O6*w*—H61⋯O4^iv^	0.83 (1)	1.99 (1)	2.821 (2)	176 (3)
O7*w*—H71⋯O2	0.84 (1)	2.05 (1)	2.874 (2)	170 (3)
O7*w*—H72⋯O5^ii^	0.83 (1)	2.33 (2)	3.034 (2)	143 (3)
O8*w*—H81⋯O2	0.83 (1)	2.07 (2)	2.854 (2)	157 (3)
O8*w*—H82⋯O3^vi^	0.84 (1)	1.96 (1)	2.799 (2)	176 (3)
O9*w*—H91⋯O5^ix^	0.83 (1)	1.95 (1)	2.747 (2)	162 (3)
O9*w*—H92⋯O6	0.83 (1)	1.97 (1)	2.795 (2)	173 (3)
O10*w*—H101⋯O8*w*	0.83 (1)	2.07 (2)	2.887 (3)	167 (4)
O10*w*—H102⋯O9*w*^vi^	0.83 (1)	2.03 (1)	2.851 (3)	171 (5)

Symmetry codes: (i) 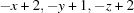; (ii) 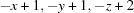; (iii) 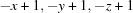; (iv) 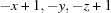; (v) 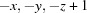; (vi) 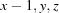; (vii) 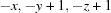; (viii) 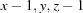; (ix) 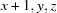.

**Fig. 1.**
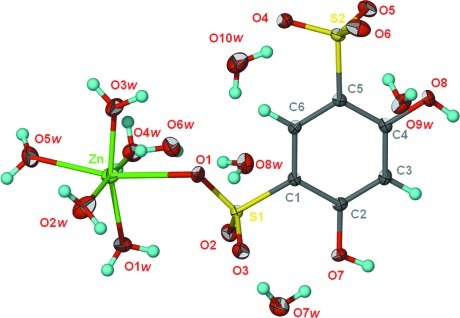
The asymmetric unit of (I) showing the atom-labelling scheme and displacement ellipsoids at the 50% probability level.

**Fig. 2.**
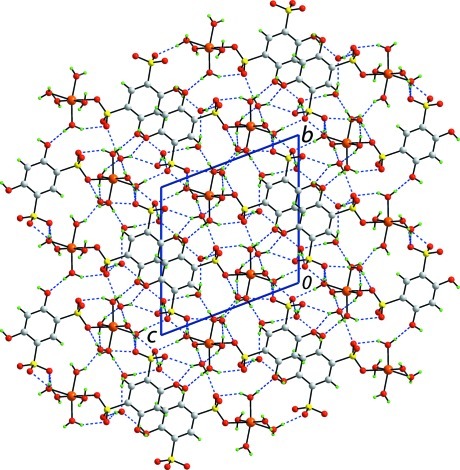
A view in projection down the a axis of the unit-cell contents of (I). The O—H···O interactions are shown as orange dashed lines.

## supplementary crystallographic information

### Comment

In earlier studies (Xie *et al.*, 2010), the crystal structure determination of [Zn(CH_3_CN)(H_2_O)_5_](C_6_H_4_O_8_S_2_).3H_2_O showed that the Zn atom is octahedrally coordinated by one acetonitrile-N atom and five water molecules. The 4,6-dihydroxybenzene-1,3-disulfonate anion did not interact directly with the metal atom, instead forming hydrogen bonds to the coordinated water molecules (Xie *et al.*, 2010). When the synthesis was repeated in the absence of acetonitrile, the title compound was obtained in which the 4,6-dihydroxybenzene-1,3-disulfonate anion is now bonded to the zinc atom, Fig. 1. The covalent Zn—O_sulfonate_ bond is relatively long compared to the Zn—O_water_ bonds, Table 1. The observed coordination geometry resembles that seen in a related pentaaquozinc/sulphonate structure (Bakirci *et al.*, 2006).

The coordinated and lattice water molecules interact with each other and with the sulphonate ligand through O—H···O hydrogen bonds to generate a tightly-held three-dimensional network, Fig. 2 and Table 2.

### Experimental

Zinc nitrate (1 mmol) and 2,4-dihydroxyl-1,5-benzenedisulfonic acid (1 mmol) were dissolved in water (10 ml). The solution was filtered and then set aside for the formation of crystals. Colourless crystals were obtained after a week.

### Refinement

Carbon-bound H-atoms were placed in calculated positions (C—H = 0.93 Å) and were included in the refinement in the riding model approximation, with *U*(H) = 1.2*U*_eq_(C). The hydroxyl H and water H atoms were located in a difference Fourier map, and were refined with the distance restraints O—H = 0.84±0.01 Å and H···H = 1.37±0.01 Å; *U*(H) = 1.5*U*_eq_(O).

### Crystal data

**Table:**

[Zn(C_6_H_4_O_8_S_2_)(H_2_O)_5_]·5H_2_O	*Z* = 2
*M**_r_* = 513.74	*F*(000) = 532
Triclinic, *P*1	*D*_x_ = 1.822 Mg m^−^^3^
Hall symbol: -P 1	Mo *K*α radiation, λ = 0.71073 Å
*a* = 7.1479 (3) Å	Cell parameters from 8162 reflections
*b* = 11.8929 (5) Å	θ = 3.0–27.5°
*c* = 12.2044 (6) Å	µ = 1.62 mm^−^^1^
α = 109.368 (1)°	*T* = 293 K
β = 104.690 (1)°	Prism, colourless
γ = 92.953 (1)°	0.21 × 0.17 × 0.14 mm
*V* = 936.27 (7) Å^3^	

### Data collection

**Table:**

Rigaku R-AXIS RAPID IP diffractometer	4257 independent reflections
Radiation source: fine-focus sealed tube	3883 reflections with *I* > 2σ(*I*)
Graphite monochromator	*R*_int_ = 0.019
ω scan	θ_max_ = 27.5°, θ_min_ = 3.0°
Absorption correction: multi-scan (*ABSCOR*; Higashi, 1995)	*h* = −8→9
*T*_min_ = 0.747, *T*_max_ = 0.820	*k* = −15→15
9270 measured reflections	*l* = −15→15

### Refinement

**Table:**

Refinement on *F*^2^	Primary atom site location: structure-invariant direct methods
Least-squares matrix: full	Secondary atom site location: difference Fourier map
*R*[*F*^2^ > 2σ(*F*^2^)] = 0.032	Hydrogen site location: inferred from neighbouring sites
*wR*(*F*^2^) = 0.091	H atoms treated by a mixture of independent and constrained refinement
*S* = 1.06	*w* = 1/[σ^2^(*F*_o_^2^) + (0.0505*P*)^2^ + 0.7681*P*] where *P* = (*F*_o_^2^ + 2*F*_c_^2^)/3
4257 reflections	(Δ/σ)_max_ < 0.001
310 parameters	Δρ_max_ = 0.67 e Å^−^^3^
32 restraints	Δρ_min_ = −0.35 e Å^−^^3^

### Special details

**Table:**

Geometry. All e.s.d.'s (except the e.s.d. in the dihedral angle between two l.s. planes) are estimated using the full covariance matrix. The cell e.s.d.'s are taken into account individually in the estimation of e.s.d.'s in distances, angles and torsion angles; correlations between e.s.d.'s in cell parameters are only used when they are defined by crystal symmetry. An approximate (isotropic) treatment of cell e.s.d.'s is used for estimating e.s.d.'s involving l.s. planes.
Refinement. Refinement of *F*^2^ against ALL reflections. The weighted *R*-factor *wR* and goodness of fit *S* are based on *F*^2^, conventional *R*-factors *R* are based on *F*, with *F* set to zero for negative *F*^2^. The threshold expression of *F*^2^ > σ(*F*^2^) is used only for calculating *R*-factors(gt) *etc*. and is not relevant to the choice of reflections for refinement. *R*-factors based on *F*^2^ are statistically about twice as large as those based on *F*, and *R*- factors based on ALL data will be even larger.

### Fractional atomic coordinates and isotropic or equivalent isotropic displacement parameters (Å^2^)

**Table:**

	*x*	*y*	*z*	*U*_iso_*/*U*_eq_	
Zn	0.13086 (4)	0.18054 (2)	0.34895 (2)	0.02620 (9)	
S1	0.51381 (7)	0.36541 (4)	0.60727 (4)	0.01787 (11)	
S2	0.58913 (7)	0.13241 (4)	0.92703 (4)	0.01968 (12)	
O1	0.4285 (2)	0.24063 (13)	0.53364 (13)	0.0256 (3)	
O2	0.3656 (2)	0.44586 (14)	0.60175 (14)	0.0260 (3)	
O3	0.6847 (2)	0.40513 (15)	0.57826 (14)	0.0272 (3)	
O4	0.4610 (3)	0.04682 (14)	0.81323 (15)	0.0335 (4)	
O5	0.5033 (2)	0.15452 (15)	1.02630 (14)	0.0277 (3)	
O6	0.7809 (2)	0.09405 (16)	0.95478 (17)	0.0331 (4)	
O7	0.7208 (3)	0.57857 (13)	0.82673 (14)	0.0294 (4)	
H7	0.777 (4)	0.637 (2)	0.8893 (18)	0.044*	
O8	0.7840 (3)	0.37304 (15)	1.10942 (14)	0.0329 (4)	
H8	0.828 (5)	0.4422 (15)	1.160 (2)	0.049*	
O1w	0.1481 (3)	0.34947 (14)	0.35174 (15)	0.0289 (3)	
H11	0.205 (4)	0.401 (2)	0.4201 (12)	0.043*	
H12	0.178 (4)	0.369 (2)	0.2982 (17)	0.043*	
O2w	0.3028 (3)	0.15031 (17)	0.24487 (19)	0.0411 (4)	
H21	0.300 (5)	0.0797 (13)	0.201 (3)	0.062*	
H22	0.412 (3)	0.191 (2)	0.263 (3)	0.062*	
O3w	0.1534 (3)	0.03068 (16)	0.38332 (19)	0.0500 (6)	
H31	0.231 (5)	0.030 (3)	0.447 (2)	0.075*	
H32	0.081 (5)	−0.0356 (19)	0.347 (3)	0.075*	
O4w	−0.0565 (2)	0.22860 (16)	0.44222 (16)	0.0313 (4)	
H41	−0.117 (4)	0.172 (2)	0.450 (3)	0.047*	
H42	−0.136 (3)	0.263 (2)	0.406 (3)	0.047*	
O5w	−0.1099 (3)	0.07189 (19)	0.18311 (16)	0.0389 (4)	
H51	−0.214 (3)	0.041 (3)	0.186 (3)	0.058*	
H52	−0.130 (4)	0.084 (3)	0.1172 (19)	0.058*	
O6w	0.6621 (3)	0.05893 (17)	0.44204 (16)	0.0330 (4)	
H61	0.623 (4)	0.025 (3)	0.3672 (10)	0.050*	
H62	0.582 (4)	0.103 (3)	0.467 (2)	0.050*	
O7w	0.3618 (3)	0.70069 (16)	0.70377 (16)	0.0320 (4)	
H71	0.369 (5)	0.6284 (12)	0.668 (2)	0.048*	
H72	0.365 (5)	0.712 (2)	0.7754 (12)	0.048*	
O8w	0.0699 (3)	0.41514 (16)	0.71292 (15)	0.0320 (4)	
H81	0.142 (3)	0.404 (3)	0.668 (2)	0.048*	
H82	−0.0465 (17)	0.409 (3)	0.673 (2)	0.048*	
O9w	1.1390 (3)	0.22383 (15)	0.98465 (17)	0.0345 (4)	
H91	1.236 (3)	0.190 (2)	0.998 (3)	0.052*	
H92	1.037 (2)	0.180 (2)	0.975 (3)	0.052*	
O10w	0.0671 (5)	0.1796 (2)	0.7321 (2)	0.0718 (8)	
H101	0.066 (8)	0.241 (2)	0.714 (4)	0.108*	
H102	0.100 (8)	0.197 (4)	0.8071 (11)	0.108*	
C1	0.5924 (3)	0.37148 (18)	0.75822 (17)	0.0186 (4)	
C2	0.6887 (3)	0.47868 (18)	0.85336 (19)	0.0207 (4)	
C3	0.7494 (3)	0.48013 (19)	0.97151 (18)	0.0231 (4)	
H3	0.8104	0.5517	1.0346	0.028*	
C4	0.7200 (3)	0.37540 (19)	0.99630 (18)	0.0218 (4)	
C5	0.6239 (3)	0.26820 (18)	0.90129 (18)	0.0195 (4)	
C6	0.5610 (3)	0.26795 (18)	0.78383 (18)	0.0200 (4)	
H6	0.4965	0.1969	0.7210	0.024*	

### Atomic displacement parameters (Å^2^)

**Table:**

	*U*^11^	*U*^22^	*U*^33^	*U*^12^	*U*^13^	*U*^23^
Zn	0.02933 (16)	0.02089 (14)	0.02952 (15)	0.00324 (11)	0.00921 (11)	0.00995 (11)
S1	0.0216 (2)	0.0159 (2)	0.0145 (2)	0.00053 (18)	0.00261 (17)	0.00571 (17)
S2	0.0248 (3)	0.0179 (2)	0.0185 (2)	0.00282 (19)	0.00699 (19)	0.00866 (18)
O1	0.0329 (8)	0.0185 (7)	0.0188 (7)	−0.0016 (6)	0.0006 (6)	0.0041 (6)
O2	0.0291 (8)	0.0240 (7)	0.0240 (8)	0.0080 (6)	0.0039 (6)	0.0096 (6)
O3	0.0283 (8)	0.0317 (8)	0.0239 (8)	−0.0009 (7)	0.0094 (6)	0.0123 (6)
O4	0.0508 (11)	0.0224 (8)	0.0213 (8)	−0.0085 (7)	0.0031 (7)	0.0076 (6)
O5	0.0330 (8)	0.0307 (8)	0.0260 (8)	0.0064 (7)	0.0148 (7)	0.0137 (7)
O6	0.0310 (9)	0.0364 (9)	0.0463 (10)	0.0153 (7)	0.0180 (8)	0.0262 (8)
O7	0.0471 (10)	0.0156 (7)	0.0209 (8)	−0.0056 (7)	0.0042 (7)	0.0064 (6)
O8	0.0491 (10)	0.0268 (8)	0.0151 (7)	−0.0062 (8)	−0.0021 (7)	0.0079 (6)
O1w	0.0389 (9)	0.0205 (7)	0.0278 (8)	0.0024 (7)	0.0081 (7)	0.0106 (6)
O2w	0.0415 (10)	0.0296 (9)	0.0476 (11)	0.0019 (8)	0.0255 (9)	−0.0010 (8)
O3w	0.0634 (14)	0.0237 (9)	0.0437 (11)	−0.0093 (9)	−0.0222 (10)	0.0183 (8)
O4w	0.0299 (8)	0.0332 (9)	0.0338 (9)	0.0037 (7)	0.0129 (7)	0.0133 (7)
O5w	0.0309 (9)	0.0531 (11)	0.0280 (9)	−0.0082 (8)	−0.0014 (7)	0.0181 (8)
O6w	0.0352 (9)	0.0363 (9)	0.0273 (8)	0.0055 (8)	0.0054 (7)	0.0137 (7)
O7w	0.0383 (9)	0.0302 (8)	0.0309 (9)	0.0087 (7)	0.0149 (7)	0.0109 (7)
O8w	0.0312 (9)	0.0354 (9)	0.0251 (8)	0.0032 (7)	0.0056 (7)	0.0073 (7)
O9w	0.0304 (9)	0.0253 (8)	0.0401 (10)	0.0006 (7)	0.0062 (8)	0.0053 (7)
O10w	0.103 (2)	0.0377 (12)	0.0521 (14)	−0.0216 (13)	−0.0148 (14)	0.0197 (11)
C1	0.0211 (9)	0.0187 (9)	0.0151 (9)	0.0011 (8)	0.0032 (7)	0.0065 (7)
C2	0.0241 (10)	0.0163 (9)	0.0209 (10)	0.0006 (8)	0.0053 (8)	0.0070 (8)
C3	0.0282 (11)	0.0184 (9)	0.0171 (9)	−0.0014 (8)	0.0024 (8)	0.0030 (7)
C4	0.0248 (10)	0.0231 (10)	0.0164 (9)	0.0014 (8)	0.0033 (8)	0.0079 (8)
C5	0.0233 (10)	0.0172 (9)	0.0178 (9)	0.0011 (8)	0.0045 (7)	0.0073 (7)
C6	0.0238 (10)	0.0153 (9)	0.0175 (9)	−0.0010 (8)	0.0025 (7)	0.0047 (7)

### Geometric parameters (Å, º)

**Table:**

Zn—O1	2.5415 (15)	O3w—H32	0.833 (10)
Zn—O1w	1.9950 (15)	O4w—H41	0.833 (10)
Zn—O2w	1.9460 (18)	O4w—H42	0.838 (10)
Zn—O3w	1.9671 (17)	O5w—H51	0.831 (10)
Zn—O4w	1.9588 (17)	O5w—H52	0.840 (10)
Zn—O5w	2.2355 (17)	O6w—H61	0.831 (10)
S1—O3	1.4528 (16)	O6w—H62	0.836 (10)
S1—O1	1.4581 (15)	O7w—H71	0.837 (10)
S1—O2	1.4675 (16)	O7w—H72	0.834 (10)
S1—C1	1.760 (2)	O8w—H81	0.832 (10)
S2—O5	1.4475 (16)	O8w—H82	0.838 (10)
S2—O4	1.4577 (17)	O9w—H91	0.828 (10)
S2—O6	1.4607 (17)	O9w—H92	0.834 (10)
S2—C5	1.761 (2)	O10w—H101	0.832 (10)
O7—C2	1.355 (2)	O10w—H102	0.834 (10)
O7—H7	0.832 (10)	C1—C6	1.388 (3)
O8—C4	1.350 (2)	C1—C2	1.404 (3)
O8—H8	0.834 (10)	C2—C3	1.390 (3)
O1w—H11	0.836 (10)	C3—C4	1.394 (3)
O1w—H12	0.832 (10)	C3—H3	0.9300
O2w—H21	0.830 (10)	C4—C5	1.403 (3)
O2w—H22	0.834 (10)	C5—C6	1.388 (3)
O3w—H31	0.836 (10)	C6—H6	0.9300
			
O2w—Zn—O4w	171.28 (8)	Zn—O2w—H22	124 (2)
O2w—Zn—O3w	95.37 (10)	H21—O2w—H22	110.9 (17)
O4w—Zn—O3w	92.91 (10)	Zn—O3w—H31	121 (2)
O2w—Zn—O1w	87.93 (8)	Zn—O3w—H32	128 (2)
O4w—Zn—O1w	84.59 (7)	H31—O3w—H32	109.9 (17)
O3w—Zn—O1w	167.14 (8)	Zn—O4w—H41	114 (2)
O2w—Zn—O5w	86.55 (8)	Zn—O4w—H42	107 (2)
O4w—Zn—O5w	91.25 (7)	H41—O4w—H42	109.4 (16)
O3w—Zn—O5w	86.37 (8)	Zn—O5w—H51	123 (2)
O1w—Zn—O5w	106.26 (7)	Zn—O5w—H52	123 (2)
O2w—Zn—O1	89.53 (8)	H51—O5w—H52	109.2 (16)
O4w—Zn—O1	95.00 (7)	H61—O6w—H62	110.4 (16)
O3w—Zn—O1	77.08 (6)	H71—O7w—H72	109.6 (16)
O1w—Zn—O1	90.55 (6)	H81—O8w—H82	110.1 (16)
O5w—Zn—O1	162.56 (7)	H91—O9w—H92	110.9 (17)
O3—S1—O1	113.06 (10)	H101—O10w—H102	111.0 (18)
O3—S1—O2	112.15 (10)	C6—C1—C2	119.17 (18)
O1—S1—O2	110.90 (9)	C6—C1—S1	119.49 (15)
O3—S1—C1	107.07 (9)	C2—C1—S1	121.33 (15)
O1—S1—C1	106.27 (9)	O7—C2—C3	121.69 (18)
O2—S1—C1	106.94 (9)	O7—C2—C1	118.50 (18)
O5—S2—O4	112.66 (10)	C3—C2—C1	119.81 (18)
O5—S2—O6	112.17 (10)	C2—C3—C4	120.69 (19)
O4—S2—O6	110.50 (11)	C2—C3—H3	119.7
O5—S2—C5	108.45 (10)	C4—C3—H3	119.7
O4—S2—C5	105.67 (10)	O8—C4—C3	121.99 (19)
O6—S2—C5	107.00 (10)	O8—C4—C5	118.46 (18)
S1—O1—Zn	123.46 (9)	C3—C4—C5	119.53 (18)
C2—O7—H7	111 (2)	C6—C5—C4	119.40 (18)
C4—O8—H8	111 (2)	C6—C5—S2	119.21 (15)
Zn—O1w—H11	114 (2)	C4—C5—S2	121.34 (15)
Zn—O1w—H12	123 (2)	C1—C6—C5	121.38 (18)
H11—O1w—H12	110.0 (16)	C1—C6—H6	119.3
Zn—O2w—H21	118 (2)	C5—C6—H6	119.3
			
O3—S1—O1—Zn	−99.48 (12)	O7—C2—C3—C4	−178.1 (2)
O2—S1—O1—Zn	27.48 (13)	C1—C2—C3—C4	1.6 (3)
C1—S1—O1—Zn	143.35 (10)	C2—C3—C4—O8	177.4 (2)
O2w—Zn—O1—S1	99.04 (12)	C2—C3—C4—C5	−1.5 (3)
O4w—Zn—O1—S1	−73.50 (12)	O8—C4—C5—C6	−178.5 (2)
O3w—Zn—O1—S1	−165.34 (14)	C3—C4—C5—C6	0.5 (3)
O1w—Zn—O1—S1	11.11 (12)	O8—C4—C5—S2	−0.8 (3)
O5w—Zn—O1—S1	175.93 (17)	C3—C4—C5—S2	178.15 (17)
O3—S1—C1—C6	−122.53 (17)	O5—S2—C5—C6	−131.59 (17)
O1—S1—C1—C6	−1.4 (2)	O4—S2—C5—C6	−10.6 (2)
O2—S1—C1—C6	117.08 (18)	O6—S2—C5—C6	107.21 (18)
O3—S1—C1—C2	56.62 (19)	O5—S2—C5—C4	50.8 (2)
O1—S1—C1—C2	177.71 (17)	O4—S2—C5—C4	171.81 (18)
O2—S1—C1—C2	−63.77 (19)	O6—S2—C5—C4	−70.4 (2)
C6—C1—C2—O7	179.0 (2)	C2—C1—C6—C5	−0.4 (3)
S1—C1—C2—O7	−0.1 (3)	S1—C1—C6—C5	178.79 (16)
C6—C1—C2—C3	−0.6 (3)	C4—C5—C6—C1	0.4 (3)
S1—C1—C2—C3	−179.75 (17)	S2—C5—C6—C1	−177.26 (16)

### Hydrogen-bond geometry (Å, º)

**Table:**

*D*—H···*A*	*D*—H	H···*A*	*D*···*A*	*D*—H···*A*
O7—H7···O9*w*^i^	0.83 (1)	1.79 (1)	2.612 (2)	170 (3)
O8—H8···O8*w*^ii^	0.83 (1)	1.84 (1)	2.664 (2)	172 (3)
O1*w*—H11···O2	0.84 (1)	2.09 (2)	2.852 (2)	151 (3)
O1*w*—H12···O7^iii^	0.83 (1)	2.09 (1)	2.923 (2)	175 (3)
O2*w*—H21···O6^iv^	0.83 (1)	2.22 (1)	3.021 (3)	162 (3)
O2*w*—H22···O7*w*^iii^	0.83 (1)	1.88 (1)	2.710 (3)	171 (4)
O3*w*—H31···O6*w*^iv^	0.84 (1)	2.01 (2)	2.765 (2)	149 (3)
O3*w*—H32···O10*w*^v^	0.83 (1)	1.79 (1)	2.618 (3)	178 (5)
O4*w*—H41···O6*w*^vi^	0.83 (1)	1.98 (1)	2.774 (2)	160 (3)
O4*w*—H42···O7*w*^vii^	0.84 (1)	1.99 (1)	2.802 (3)	164 (3)
O5*w*—H51···O4^v^	0.83 (1)	2.00 (1)	2.833 (3)	176 (4)
O5*w*—H52···O6^viii^	0.84 (1)	1.97 (1)	2.802 (3)	170 (3)
O6*w*—H62···O1	0.84 (1)	2.08 (1)	2.903 (2)	168 (3)
O6*w*—H61···O4^iv^	0.83 (1)	1.99 (1)	2.821 (2)	176 (3)
O7*w*—H71···O2	0.84 (1)	2.05 (1)	2.874 (2)	170 (3)
O7*w*—H72···O5^ii^	0.83 (1)	2.33 (2)	3.034 (2)	143 (3)
O8*w*—H81···O2	0.83 (1)	2.07 (2)	2.854 (2)	157 (3)
O8*w*—H82···O3^vi^	0.84 (1)	1.96 (1)	2.799 (2)	176 (3)
O9*w*—H91···O5^ix^	0.83 (1)	1.95 (1)	2.747 (2)	162 (3)
O9*w*—H92···O6	0.83 (1)	1.97 (1)	2.795 (2)	173 (3)
O10*w*—H101···O8*w*	0.83 (1)	2.07 (2)	2.887 (3)	167 (4)
O10*w*—H102···O9*w*^vi^	0.83 (1)	2.03 (1)	2.851 (3)	171 (5)

Symmetry codes: (i) −*x*+2, −*y*+1, −*z*+2; (ii) −*x*+1, −*y*+1, −*z*+2; (iii) −*x*+1, −*y*+1, −*z*+1; (iv) −*x*+1, −*y*, −*z*+1; (v) −*x*, −*y*, −*z*+1; (vi) *x*−1, *y*, *z*; (vii) −*x*, −*y*+1, −*z*+1; (viii) *x*−1, *y*, *z*−1; (ix) *x*+1, *y*, *z*.
